# A Rare Case of Nutcracker Phenomenon Associated With Midgut Malrotation and Congenital Solitary Kidney

**DOI:** 10.7759/cureus.49653

**Published:** 2023-11-29

**Authors:** Atta Ur Rehman, Muhammad Haris Latif, Ali Haider, Emaan Mazhar, Bahar Bastani

**Affiliations:** 1 Internal Medicine, Nishtar Medical University, Multan, PAK; 2 Nephrology, Saint Louis University School of Medicine, St. Louis, USA

**Keywords:** hyperuricemia, proteinuria, hematuria, hypertension, nutcracker phenomenon, congenital solitary kidney, midgut malrotation, nutcracker syndrome

## Abstract

Medical conditions such as the nutcracker phenomenon, midgut malrotation, and congenital solitary kidney are rare. Hereby, we present a 21-year-old South Asian male referred to the nephrology clinic for hypertension and increased serum creatinine and was diagnosed with all three conditions. We briefly reviewed the literature on this subject. We present this case report to highlight the complex interplay of multiple renal and gastrointestinal anomalies and to emphasize the importance of a multidisciplinary approach in patient care to optimize outcomes and quality of life.

## Introduction

The "nutcracker phenomenon" (NCP) occurs when the left renal vein (LRV) is compressed between the aorta and the superior mesenteric artery (SMA) [1]. When patients have clinical symptoms and signs, it is called nutcracker syndrome (NCS) [2]. Although an aortomesenteric (AM) angle of less than 35-39 degrees is agreed upon to be required for the syndrome to manifest, NCS is still considered to be a diagnosis of exclusion. Therefore, imaging and physiological metrics are used to confirm the diagnosis. The peak age of incidence of NCS is in the second decade to middle age, although it ranges from childhood to the seventh decade. The exact prevalence of NCS is unknown, but recent studies suggest that gender predilection is equal between males and females [3].

Regarding the physical features predisposing patients to the NCS, intestinal malrotation, a congenital disability caused by improper rotation and fixation of the midgut during development, is a potential etiology [4]. Patients may be predisposed to developing NCS because of a lean body habitus, rapid weight loss, and incorrect intestine configuration, particularly right-side duodenal misalignment in which the duodenum does not pass through the aortomesenteric angle. While often detected in infancy, undiagnosed malrotation in adulthood cases has been reported, often accompanied by vague symptoms and diagnostic challenges.

Unilateral Renal Agenesis is a rare phenomenon with varying degrees of clinical impact. Individuals with this condition may lead relatively normal lives, with the remaining kidney compensating for the absent one. However, early detection and management are crucial to prevent potential complications like hypertension, proteinuria, and chronic kidney disease. If unilateral renal agenesis is suspected, the possibility of a pelvic or ectopic kidney should be assessed. Hypertrophy of the visualized kidney suggests unilateral renal agenesis [5].

## Case presentation

A 21-year-old South Asian male was referred to our nephrology clinic in 2019 to evaluate hypertension and increase Serum Creatinine from 1.0 to 1.21 mg/dl. He denied experiencing chest pain, shortness of breath, dyspnea on exertion, cough, wheezing, abdominal/flank pain, dysuria, or hematuria. The patient had no symptoms at that time. However, he had been known to have hypertension, for which he was referred to our clinic.

**Figure 1:**
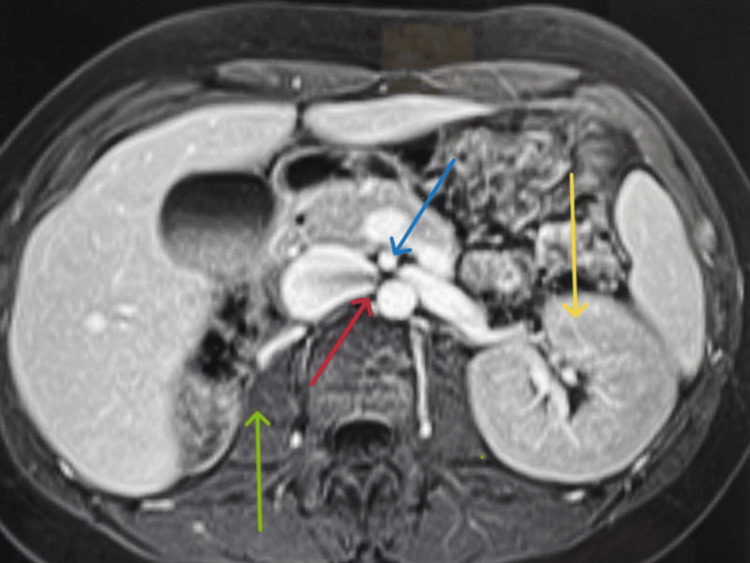
MRA: Cross-sectional view The Blue Arrow shows a Superior Mesenteric artery originating from the abdominal aorta. The Red Arrow shows a narrowing of the Left renal vein by a superior mesenteric artery. The Yellow Arrow shows the left Kidney. The Green Arrow shows an absent right Kidney. Abdominal MRA had demonstrated the absence of the ascending colon in the right retroperitoneal area and the absence of the duodenum passing through the aortomesenteric portion. Most parts of the small intestine were located on the right side of the abdomen, with the colon predominantly positioned on the left hemiabdomen, indicative of intestinal malrotation.

**Figure 2:**
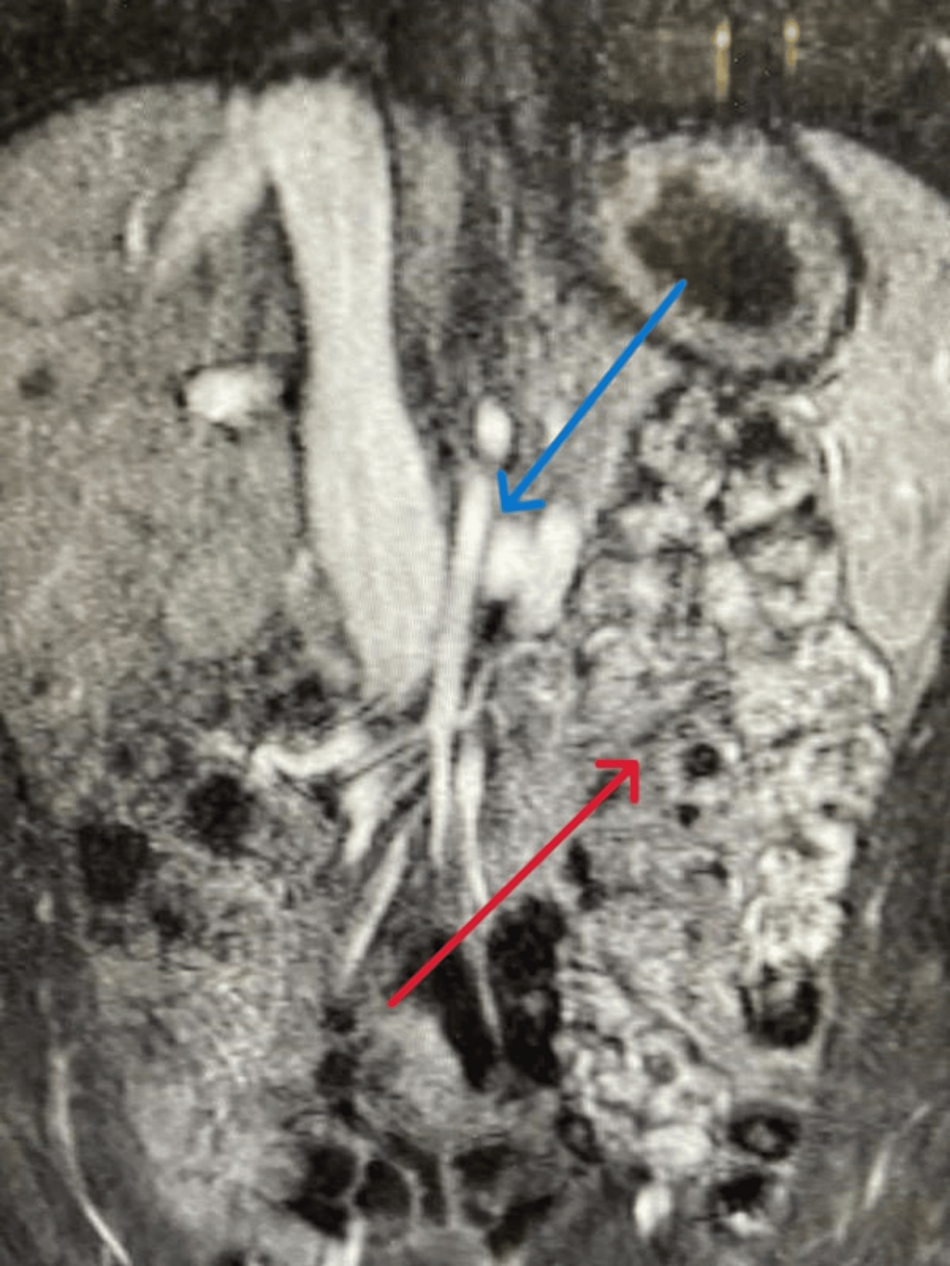
Magnetic resonance angiography Blue Arrow shows the Superior mesenteric artery compressing the left renal vein. Red Arrow shows intestinal malrotation.

The patient's medical history was significant for congenital solitary kidney and intestinal malrotation, as noted on previous imaging studies (Doppler USG and MRA) performed on 10/10/2014 to investigate the cause of his hypertension. Abdominal MRA (Figure 1) has demonstrated the absence of the ascending colon in the right retroperitoneal area and the absence of the duodenum passing through the aortomesenteric portion. Most parts of the small intestine were located on the right side of the abdomen, with the colon predominantly positioned on the left hemiabdomen, indicative of intestinal malrotation (Figure 2). Further, the imaging findings confirmed the absence of the right kidney and showed a normal left kidney and renal artery flow. A dilated left renal vein was noted to be compressed as it crossed the midline between the aorta and superior mesenteric artery, consistent with the nutcracker phenomenon. It also showed an accessory renal artery.

Abdominal ultrasonography on 11/27/2019 revealed a solitary left renal kidney measuring 13.5x6.8x6.0 cm with a volume of 290 ml and normal renal parenchymal echogenicity. Mild collecting system dilation was also noted. Apart from this, there was no other pathology noted. Further investigation to rule out other secondary causes of hypertension

On physical examination, the patient's body weight, height, and body mass index were within normal limits. His blood pressure was 120/57, pulse was 69, and respiratory rate was 17 (Table 1). His home blood pressure readings consistently showed higher values in the 140s/70s. He had been on antihypertensive therapy since 2014. Importantly, we systematically investigated common secondary causes of hypertension, such as renal artery stenosis and primary hyperaldosteronism, all of which were ruled out through clinical examination and laboratory assessments. Furthermore, the patient had no family history of hypertension, indicating a non-hereditary predisposition to the condition. These findings allowed us to focus on the unique aspects of the patient's medical history, including congenital anatomical variations, to understand the underlying mechanisms of his hypertension better.

**Table 1:** Physical exam findings and vitals

Weight kg	Height cm	BMI kg/m2	Blood pressure mmhg	Respiratory rate/ minute	Pule/minute
76	183	22.7	120/57	17	69

Laboratory investigations and urine analysis (Table 2-4) revealed a serum creatinine level of 1.21 mg/dl, an estimated glomerular filtration rate (eGFR) ranging in the 80s ml/min/1.73m², random urine protein to creatinine ratio of 329 mg/g, and uric acid of 6.5 mg/dl.

**Table 2:** Laboratory investigations

Relevant Laboratory investigations	08/02/23	02/06/23	12/03/20	08/10/20	01/25/20	Reference values
Creatinine (mg/dL)		1.21	1.06	1.10	1.06	0.7-1.3
Blood urea nitrogen (mg/dL)		20	16	17	18	7-18
Uric Acid (mg/dL)		6.5	6.1	5.7	-	3.5-7.4
Effective Glomerular filtration rate (ml/min/1.73m)		85	101	97	102	90-120
Urine Protein: Creatinine (mg/g)	100	329	483	281	170	<200
Plasma Renin (mcg/L/Hr)	4.3					0.6-4.3
Plasma Aldosterone (ng/dL)	24					2-9
Bicarbonate mEq/L		30	31	31	31	22-29
Potassium mEq/L		4.7	4.6	4.4	4.1	3.5-5.2
Phosphate mEq/L		4.2	3.6	3.9		2.5-4.5
Sodium mEq/L		137	140	140	137	135-145

**Table 3:** Urine Analysis

Colour	Pale Yellow
Appearance	Clear
Specific gravity	1.015
pH	6.5
Leukocytes	negative
Nitrite	negative
Protein	++
Glucose	negative
Ketones	negative
Blood	negative

**Table 4:** Complete blood count MCH: mean corpuscular hemoglobin; MCHC: Mean corpuscular hemoglobin concentration; RDW: red cell distribution width; ESR: erythrocyte sedimentation rate; MCV: mean corpuscular volume

Hemoglobin g/dl	12
RBC 10^6 u/ml	5.2
Hematocrit %	38
MCH pg	28
MCHC g/dl	33
RDW %	12
WBC 10^3 u/ml	6.7
Neutrophil %	60
Monocyte %	8
Esosiophil %	2
Basophil %	0
Lymphocyte %	30
Platelet 10^3 u/ml	256
ESR mm/hr	2
MCV fl	83

Management and follow-up

The patient was initially managed conservatively with 100 mg Lorsartan and 5 mg Amlodipine to control blood pressure. The patient's SCr (serum creatinine) levels remained relatively stable during subsequent follow-ups, ranging from 1.04 to 1.21. However, there was an increase in urine protein levels from 172 to 483; subsequently, Amlodipine was switched to 240 mg Diltiazem for its anit-proteinuric effects, which successfully lowered the urine protein levels to 329 mg/g. Uric acid levels also increased from 5.7 to 6.5, for which 100 mg of Allopurinol was prescribed to manage the elevated uric acid levels. Fortunately, the patient's blood pressure remained stable throughout this period. Considering the patient's symptoms and potential complications associated with the nutcracker phenomenon, the possibility of future endovascular stenting was discussed as a treatment option. Regular follow-up appointments were scheduled every three months to monitor symptom progression and assess the need for further interventions.

## Discussion

Nutcracker phenomenon (NCS) is a rare condition, and its coexistence with intestinal malrotation and congenital solitary kidney is exceptionally uncommon. The exact prevalence of NCS is unknown. While intestinal malrotation has been identified as a predisposing factor for the nutcracker phenomenon, further research is needed to determine if having a solitary kidney also contributes to its development. Unilateral renal agenesis is estimated to be between 1/2500 and 1/4000 live births [6]. Intestinal malrotation occurs between 1 in 200 and 1 in 500 live births. The association between the solitary kidney and the nutcracker phenomenon raises intriguing questions about potential underlying mechanisms.

To the best of our knowledge, this is the first reported case of the nutcracker phenomenon coexisting with intestinal malrotation and congenital solitary kidney. Only one previous case of the nutcracker phenomenon coexisting with intestinal rotation has been documented in the PubMed database [7,8]. While NCS is frequently considered idiopathic, various diseases and conditions can predispose individuals to its development. These include pancreatic neoplasm, para-aortic lymphadenopathy, retroperitoneal tumor, abdominal aortic aneurysm, and diminished retroperitoneal and mesenteric fat, all of which may cause the aortomesenteric angle to narrow, compressing the left renal vein (LRV). Similarly, our patient, who has a tall and lean physical habitus, might have developed a nutcracker phenomenon during his growth spurt, supporting that diminished retroperitoneal fat predisposes to the nutcracker phenomenon. Treating the underlying causes of these diseases and illnesses may be necessary to resolve NCS effectively. Recognizing the underlying etiologies is crucial in optimizing therapeutic approaches, given the potential adverse consequences of NCS, such as chronic renal disease due to increased LRV pressure or LRV thrombosis [2].

Symptoms of patients with nutcracker syndrome may include hematuria (78.57%), left flank pain (38.39%), varicocele in males (35.71%), proteinuria (30.36%), and anemia (13.39%) [4,9]. However, these findings were absent in our patient. Although hypertension is not a classic sign of the Nutcracker syndrome, a few reports have described its association [10]. Our case report adds to the existing literature by presenting a young man with hypertension and hyperuricemia accompanying the Nutcracker phenomenon.

In evaluating this unique case, it is imperative to acknowledge the importance of excluding common secondary causes of hypertension. Secondary hypertension is typically attributed to identifiable underlying conditions that contribute to elevated blood pressure. Common etiologies include renal artery stenosis, primary hyperaldosteronism, and pheochromocytoma, among others. In our patient, thorough clinical examination and laboratory assessments ruled out these well-recognized causes of secondary hypertension. Additionally, the absence of a family history of hypertension suggests a non-hereditary predisposition to the condition. This comprehensive assessment allowed us to focus on the exceptional aspects of the patient's medical history, including congenital anatomical variations, notably the presence of the nutcracker phenomenon, intestinal malrotation, and a congenital solitary kidney. By excluding these common causes, we could explore the potential link between these anatomical anomalies and the development of hypertension, offering a unique perspective on the pathophysiology of this complex case.

Our literature review revealed that hypertension in patients with NCS could be due to increased renin-aldosterone levels, supported by a case report in which a patient with NCS presented with hypertension. The patient had elevated renin and aldosterone levels, which decreased significantly after being treated with left renal vein stent placement [11]. However, we also came across a study in which two patients with NCS also presented with hypertension, but their renin-aldosterone levels were normal [12]. 

This raises the question of whether or not NCS leads to increased renin-aldosterone levels and subsequent hypertension. Further research is required to make this more clear. We could not find any other pathology in our patient that would cause hypertension, leading to the assumption that the Nutcracker phenomenon might cause it.

While our case primarily focuses on the coexistence of the Nutcracker phenomenon, intestinal malrotation, and congenital solitary kidney, it's essential to briefly address intestinal malrotation and its potential clinical implications. Midgut malrotation is a congenital anomaly that can lead to severe complications, such as midgut volvulus, a life-threatening condition. In our patient's case, he had a history of intestinal malrotation, as noted in previous imaging studies. While midgut volvulus did not manifest in our patient, it is crucial to highlight the importance of early recognition and surgical intervention in cases of malrotation to prevent potentially catastrophic complications. The definitive surgical management for midgut malrotation is Ladd's procedure, which involves derotation of the malrotated bowel and dividing Ladd's bands. Although it was not indicated in our patient's case, understanding the clinical implications and treatment considerations associated with midgut malrotation, including Ladd's procedure, is vital for medical practitioners and readers to appreciate the significance of early diagnosis and intervention in preventing life-threatening complications.

While this case report offers valuable insights, we acknowledge its limitations, including the retrospective nature and focus on a single patient. A more extensive case series or prospective studies involving patients with similar conditions may provide a better understanding of the clinical spectrum and therapeutic strategies for this condition.

## Conclusions

This case report highlights the rare presentation of the nutcracker phenomenon in association with unilateral renal agenesis and Midgut Malrotation. The clinical implications of this co-occurrence warrant further investigation. Healthcare providers should be aware of the possibility of the nutcracker phenomenon in patients with congenital solitary kidneys who present with hypertension, proteinuria, and hyperuricemia. Early diagnosis and a multidisciplinary approach are essential for appropriate management and improved patient outcomes.
